# Association between CT-Quantified Body Composition and Recurrence, Survival in Nonmetastasis Colorectal Cancer Patients Underwent Regular Chemotherapy after Surgery

**DOI:** 10.1155/2021/6657566

**Published:** 2021-03-25

**Authors:** Piaopaio Ying, Wenyi Jin, Xiaoli Wu, Weiyang Cai

**Affiliations:** ^1^Department of Pneumology, The First Affiliated Hospital of Wenzhou Medical University, Wenzhou, China; ^2^Department of Gastroenterology, The First Affiliated Hospital of Wenzhou Medical University, Wenzhou, China

## Abstract

**Background:**

Body mass index, measured at colorectal cancer (CRC) diagnosis has been associated with recurrence and survival outcomes. Computed tomography- (CT-) defined body compositions accurately reflect body mass, but there was no consistent perspective on the influence of visceral adipose tissue (VAT) and skeletal muscle mass (SM) on the prognosis of nonmetastasis CRC, especially in the patients underwent surgery and regularly standard chemotherapy.

**Methods:**

We investigated the associations of CT-quantified body composition (VAT and SM) with CRC patients successively underwent surgery and regular 8-12 of periods standard chemotherapy. All of the CT images were obtained at the level of the L3/4 spinal level. The prognostic value of the body compositions was analyzed using the Cox regression model, and precise clinical nomograms were established.

**Results:**

In XELOX-treated patients, progression-free survival (PFS) (*P* = 0.025) and overall survival (OS) (*P* = 0.032) were lower in the high-SM than in the low-SM group. The univariate analysis demonstrated that compared with low-SM patients, patients with high-SM showed a strikingly poor prognosis in both OS (*P* = 0.0512) and PFS in the T4 subgroup (*P* = 0.0417), while contrary to the T2-3 subgroup.

**Conclusions:**

CT-quantified body compositions have a significant influence on CRC patients successively underwent curative resection and regularly standard chemotherapy with the endpoints of 1-year, 3-year, and 5-year both OS and PFS. Patients with high-SM showed a strikingly poor prognosis in OS and PFS in the T4 subgroup; however, the prognosis role of body composition was opposite in T2-3 patients.

## 1. Introduction

Currently, colorectal cancer has a high incidence rate and is the secondary causation of cancer mortality worldwide, extremely posing a threat to human health [1, 2]. Therefore, it is necessary to identify the prognostic factors of progression-free survival (PFS) and overall survival (OS) in the early time and take effective and targeted interventions to improve the prognosis. According to previous literature, overweight and obesity were found associated with recurrence-free (RF) and survival among colorectal cancer patients. However, conflicting results between them are observed in different studies. Besides, traditional index, such as body mass index (BMI), is insufficient for reflecting the distinguishing between fat and muscle mass or visceral adipose tissue (VAT) and skeletal muscle (SM) [3]. Abdominopelvic computed tomography (CT) imaging is not only a routine examination as a pretreatment staging way of clinical management in cancer patients but also a more accurate method to differentiate body composition, which means that no further test exposure is required, and no additional financial burden is placed on the patient. Moreover, CT-defined body composition is widely confirmed for accurate reflecting on different types of adipose tissue as well as muscle mass [4].

The rapid growth and proliferation of tumor cells require a large amount of energy, which is a key factor resulting in high death from tumors. Based on existing researches, baseline fat and muscle distribution are closely related to postoperation recovery and complications, tolerance of chemotherapy-induced toxicity, and recurrence as well as health-related quality of life [5–7]. Therefore, we speculate that body compositions may play a role in nonmetastasis colorectal cancer patients. Although it is acknowledged that body compartments are independent prognostic predictors, however, there was no consistent perspective on the influence on VAT and SM to the prognosis of nonmetastasis colorectal cancer, especially in the patients who successively underwent curative resection and regularly standard chemotherapy [8, 9]. Moreover, the general consensus demonstrated that the prognosis of colorectal cancer was closely associated with the staging features of TNM classification, including pathologic T stage, absence, of nodal involvement, with or without distance. Worse prognosis in these colorectal cancer patients with distant metastases, thus, we did not include these patients to avoid bias in the results of our research. In addition, previous researches about the relationship between body compositions and the prognosis of CRC, most of which were based on the CRC stage or not, there was a lack of dividing T stage into two groups to explore the connection between them [10].

Our study is aimed at exploring the relationship between CT-quantified body compositions and recurrence as well as the overall survival of colorectal cancer patients who successfully underwent curative resection and had regular 8-12 periods of standard chemotherapy. Also, the independent risk factors in these patients were analyzed. Thus, we try to construct the OS and RF nomogram to predict the 1-year, 3-year, and 5-year survival probability based on the prognostic factors derived from multivariate Cox regression analysis.

## 2. Methods

### 2.1. Study Population and Design

The records of 221 persons diagnosed with CRC at the First Affiliated Hospital of Wenzhou Medical University between January 2014 and January 2017 were reviewed. Eligible patients were defined as firstly diagnosed with primary colorectal cancer, excluded other malignant tumors, 18 years or over, stage I–III, complete pathology, laboratory, and able to provide informed consent. Simultaneously, all of the patients successively underwent surgery and regular 8-12 periods of standard chemotherapy (including XELOX and FOLFIRI/FOLFOX). Patients who did not undergo adequate abdominopelvic computed tomography scanning before starting surgery, those treated for irregular or no chemotherapy, and those who lose followed-up for <24 months were excluded. Pathologists assessed the tumor stage according to the 8th edition of the AJCC TNM staging guidelines. In general, there were 221 eligible cases selected in this study, and the pathological T stages were T2-4. All of these patients were followed up, and 91 recurrent and 66 dead patients were recorded during the follow-up. According to the American Joint Committee on Cancer (AJCC) TNM (Tumor, Nodes, Metastasis) system and the staging 8th edition of colorectal cancer, pathologic T4 stage was defined as tumor invasion of the visceral peritoneum or adherences to adjacent organ or structure. The deep tumor penetration and invasion of adjacent organs were extremely related to the risk of relapse and overall survival among CRC patients without distant metastasis. Based on many previous studies, T4 had a significant impact on affecting both the duration and effect of chemotherapy [11]. Meanwhile, combined with other existing researches on the grouping of T staging [12], we divided all patients into T2-3 and T4 groups. The cutoff time of the study was set in August 2020. The study protocols were approved by the Wenzhou Medical University Ethics Committee. All procedures adhere to the BRISQ Guidelines for reporting research on human biospecimens.

### 2.2. Body Composition

**Figure 1:**
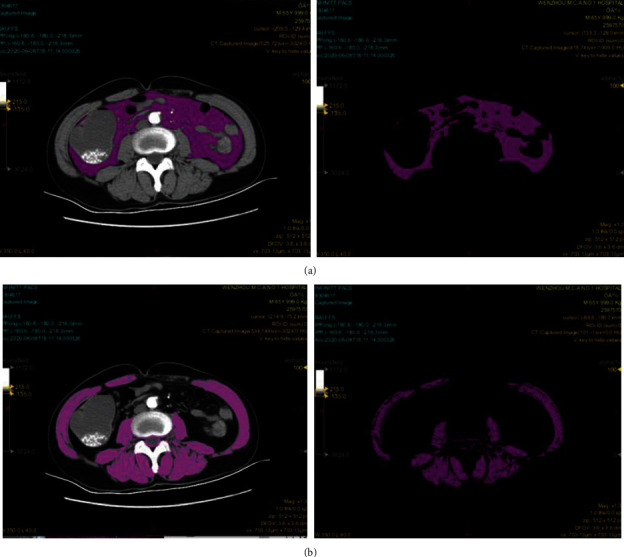
Example of a computed tomography (CT) scan with the area-based, densitometric quantification of adipose tissue (threshold: −190 to −30 HU) measured at spinal level L3/4: regions of interest (ROI) containing visceral fat area (VAT) (a) and an example of the densitometric quantification of muscle area (SM), dorsal and psoas muscles (threshold: 40 to 100 HU) (b).

Muscle mass and visceral fat mass were evaluated using pretreatment CT images obtained at the level of the L3/4 spinal level in detail [13]. Patients all underwent multidetector CT scans with quantification of body composition within 15 days before surgery. Specific regions of interest (ROI) were manually determined: VAT (by defining the fascial plane of the abdominal muscle wall, using standard Hounsfield Unit (HU) ranges adipose tissue -190 to -30, Figure 1(a)) and SM (by defining the skeletal muscle using HU ranges muscle tissue 40 to 100, Figure 1(b)). All CT examinations were performed using the scanners: Brilliance-64, Philips Medical Systems, Eindhoven, The Netherlands; 128-MDCT scanner Somatom Definition, Siemens Health-care Sector, Forchheim, Germany. Two experienced radiologists drew the eligible CT planar, and then, CT analysis of the contrast-enhanced CT images was performed using LifeX software. To assess accuracy, two individuals performed scan measurements.

### 2.3. Body Mass Index

Patients were categorized according to their Eastern Cooperative Performance Status (ECOG-PS) into five district grades (grades 0–4) and assessed by either the treating clinician or clinical research staff. In this analysis, we gathered grade 0 as ECOG-1, grades 1-2 as ECOG-, and grades 3-4 as ECOG-3. The metabolic syndrome was internationally defined as included more than three criteria: (1) BMI was greater than 25.0 kg/m^2^; (2) diagnosed with diabetes; (3) diagnosed with hypertension SBP/DBP > 140/90 mmHg; (4) blood HDL − C < 0.9 mmol/L; (5) blood TG > 1.7 mmol/L.

### 2.4. Statistical Analyses

R software, GraphPad Prism, and Stats were conducted for statistical analyses. The OS and PFS nomogram were constructed based on the prognostic factors derived from multivariate Cox regression analysis to predict 1-, 3-, and 5-year survival possibilities. Continuous variables were exhibited for means, medians, range, and standard deviation (SD) and compared using an independent *t*-test or Wilcoxon test; Spearman' correlation coefficient was used for variable correlation; Chi-square test was used to analyze categorical variables; log-rank survival analysis was employed to determine the effect of various variables on patient OS and PFS. All statistical tests were two-sided and *P* < 0.05 was considered statistically significant.

## 3. Results

### 3.1. Participators Characteristics

Of 221 eligible colorectal cancer patients, who were treated with surgery and periodic chemotherapy, were recruited from Wenzhou Medical University from 2014 January 1st to 2017 January 1st. Other CRC patients were excluded from the current analysis for having incomplete or no CT-based body compositions quantification or irregular chemotherapy. In our study, the proportion of patients with hypertension, diabetes, and MetS was 36.5%, 30.8%, and 12.1%, respectively. As of August 2020, 63 patients died during follow-up, none lost follow-up. Baseline clinicopathological parameters were presented in Table 1. As for the associations of adipose and muscle tissue with health-related index, we observed that SM was closely associated with ECOG and Mets score in T2-3 CRC subgroups (Table S1-S2).

Of 221 eligible patients who were diagnosed with CRC, 179 received XELOX chemotherapy and 42 received FOLFIRI/FOLFOX as first-line treatment for CRC (Table 1). The median age was 60.66 ± 12.45 months in patients treated with XELOX and 61.17 ± 16.29 months in those treated with FOLFIRI/FOLFOX. Baseline characteristics were similar between XELOX-treated and FOLFIRI/FOLFOX-treated patients except for BMI and ECOG-PS score, which was similar within the two groups.

### 3.2. Impact of Body Composition on Survival in CRC Patients with T Stage

**Figure 2:**
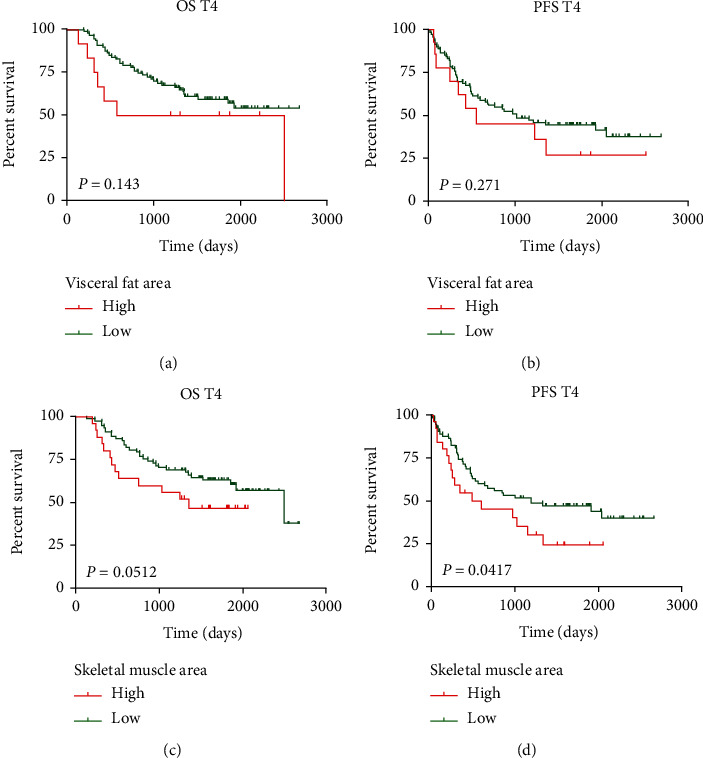
Outcomes T4 CRC patients based on CT body composition. Outcomes based on visceral fat in T4 GC patients from the time of diagnosis. (a) OS; (b) PFS; outcomes based on skeletal muscle in T4 GC patients from the time of diagnosis. (c) OS; (d) PFS.

The average median VAT and SM were 8.852 and 6.504, respectively. T4 stage was defined as penetrating the visceral peritoneum or directly invading or adhering to other organs or structures according to the 8th edition of the AJCC TNM staging guidelines. The degree of penetration of the tumor through the bowel wall or adhere to adjacent organs or structures played a crucial role in the prognosis of CRC. In present proof-of studies had found that the tumor stage (T4) was an independent risk factor for recurrence and OS [11, 14]. Thus, we divided the patients into *T* < 4, and T4 in the cohort. Recurrence and overall survival outcomes of <T4 and T4 patients basing on VAT and SM were shown, respectively, in Figure S1 and Figure 2. We surprisingly found that the prognosis roles of body compositions seem to be opposite in these two subgroups. Especially, the univariate analysis demonstrated that compared with low-SM patients, patients with high-SM showed a strikingly poor prognosis in both OS (*P* = 0.0512, Figure 2(c)) and PFS in the T4 subgroup (*P* = 0.0417, Figure 2(d)). Time on the T2-3 subgroup was shorter for the low group than for the high group, although the difference was not statistically significant in OS and PFS (Figure S1).

### 3.3. Body Composition and Use of Chemotherapy

Totally, 179 and 42 CRC patients were treated with XELOX- and FOLFIRI/FOLFOX-treated chemotherapy, respectively, with a median of eight and twelve treatment cycles. In XELOX-treated patients, patients PFS (*P* = 0.025; Figure S2C) and OS survival (*P* = 0.032; Figure S2D) were lower in the high-SM than in the low-SM group. In FOLFIRI/FOLFOX-treated patients, overall survival (*P* = 0.2108 and 0.2701; Figure S3A and S3C), and progression-free survival (*P* = 0.6163 and 0.8542; Figure S3B and S3D) were similar between the VAT and SM high and low groups.

### 3.4. Construction of the CT-Based Nomogram

**Figure 3:**
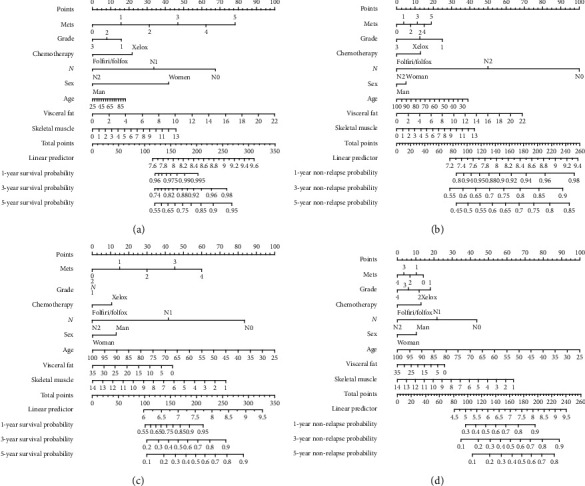
The nomogram to predict the 1-, 3-, and 5-year overall survival (a) and progression-free survival rates (b) of T2-T3 CRC patients. The nomogram to predict the 1-, 3-, and 5-year overall survival (c) and progression-free survival rates (d) of T4 CRC patients.

To establish a clinically applicable method for predicting the prognosis of CRC patients, we next established a prognostic nomogram to predict the survival probability at 1, 3, and 5 years for XELOX patients. Some independent prognostic parameters, including Mets, chemotherapy, grade, N stage, Age, VAT, and SM, were enrolled in the prediction model (Figure 3). As shown in Figure 3, the VAT and SM contributed the most risk points in CRC patients, whereas the other clinical factors contributed much less. In advanced malignant CRC patients, patients PFS and OS were higher in low-SM and VAT than in the high-SM and VAT group (Figures 3(c) and 3(d)). The trend was totally reversed in the <T3 subgroup (Figures 3(a) and 3(b)). In general, the VAT and SM were independent risk predictors for the survival of CRC patients.

## 4. Discussion

Body mass index has been found associated with CRC postoperative complications and survival outcomes. However, traditional indexes, such as Mets, BMI, waist/hip ratio, and ECOG-PS, do not provide detailed quantitative data for clinical reference [15]. Recently, body composition has appeared as a substitution to the traditional index. Body compositions, including skeletal muscle and visceral fat, can be estimated easily and accurately using CT images and software programs. Based on existing studies, pathological T staging was extremely associated with the duration of postoperative adjuvant chemotherapy. Furthermore, the T4 stage was the critical prognostic factor of tumor recurrence and overall survival. To our knowledge, this was the first literature to divide into subgroups according to T staging and investigate the association between body composition and disease progression, mortality, and efficacy of first-line treatment in nonmetastasis CRC patients who underwent regular chemotherapy after surgery in CT-based parameters manner. Besides, CT-quantified body composition nomograms have not systematically been estimated in CRC patients who successfully underwent curative resection and had regular standard chemotherapy.

According to the nomogram, SM and VAT played a dominant role in the prognosis of CRC patients, especially in T4 patients. We surprisingly found some conclusions worthy of consideration. High VAT and high SM were poor prognosis of RF and OS in the T4 subgroup while were protective prognosis in the T2-3 subgroup. On the other hand, body composition was associated with the risk of not only survival rate, but also chemotherapy toxicity. XELOX and FOLFOX/FOLFIRI are the most widely used first-line chemotherapy in patients with CRC. Although chemotherapy treatments have demonstrated a survival benefit and are widely approved for clinical use for CRC patients, the optimal treatment strategy remains to be determined. The therapeutic effects are unquestionably valid for patients, however, whether it can work on different physiological features of CRC patients is still controversial and has not been reported to date. Therefore, predictive markers of survival and treatment response in CRC are critically needed. In XELOX-treated patients, the high-SM group had lower progression-free survival (*P* = 0.025) and overall survival (*P* = 0.032) than the low-SM group. Given the limited number of patients, we have not explored the associated risk of CRC progression, radiologic progression, and overall mortality in FOLFOX/FOLFIRI-treated patients.

As was showed in Figure S1, lower VAT and SM were meaningful risk factors in CRC recurrence and overall survival among the T2-3 stages of CRC patients received regular chemotherapy after curative surgery, consistent with the view that lacking muscle mass had adverse consequences early in the malignant tumor. We speculate that low-VAT and low-SM may be related to higher chemotherapy toxicity, especially in the early stage [16]. Besides, there may be an extreme loss of fat and muscle tissue during not only the development of cancer but also the chemotherapy process [17]. T2-3 stages of CRC patients have longer OS than the T4 subgroup, higher VAT, and SM provided fat tissue and muscle tissue to be consumed and was easier to tolerate the side effects of chemotherapy, resulting in slower developing a state of cachexia and confer a survival advantage. Moreover, fewer myokines and interleukin were released as a consequence of low SM, leading to an imbalance of the immune system and having a poor impact on prognosis in the lower T stage [18]. Thus, targeted and preventive intervention strategies such as nutritional intervention and physical exercise should be given for CRC T2-3 stage patients, avoiding developing sarcopenia, thereupon then decreasing the recrudescence and overall survival rate [19, 20]. However, due to skeletal muscle did not effectively reflect muscle function, CT-based body components combined with functional measures assessed by handgrip strength and stair-climbing power would be more precise [21].

As for CRC T4 stage patients, in our research, low VAT and low SM were propitious to have a survival benefit; in addition, visceral fat had a greater impact than muscle, contradicted with T2-3 stages. The reasons why gave rise to the opposite phenomenon deserved deeper investigation. Inferring that most of the T4 stage CRC patients were at the state of cachexia, a multifactorial paraneoplastic syndrome characterized by carbohydrate, lipid, and protein metabolic disturbance, inflammatory and immunocompromised status, and higher VAT and SM would be a burden for advanced cancer patients, rather than an advantage [22]. CRC patients of the T4 stage, the higher VAT, and SM, the more enhanced inflammation, and hypermetabolism entered a vicious cycle, ending up in refractory cachexia [23]. Advanced cachexia, nevertheless, was extremely difficult to redress for drugs, nutrition intake, and physical exercise according to existing studies. The human body reached a relative metabolic balance via abatement lipolysis and proteolysis [24]. Furthermore, high visceral fat was inclined to cause insulin resistance, which was a risk factor for cancer progression [25]. Adiposity as well as a metabolic disorder was susceptible to infection and other complications, which were contributors to shortened progression-free survival and overall survival. Moreover, immune cell expression and secretion were induced by cancer; in the pathogenesis of a malignant tumor, more fat and muscle would provide a better environment, resulting in the suppression of immune and chronic inflammation.

FOLFIRI/FOLFOX-treated was a strong combination chemotherapy regimen, and the adverse reactions caused by chemotherapy were also stronger. Patients with poor physical condition and intolerance to strong combination chemotherapy regimens were more inclined to choose the XELOX-treated regimen. Therefore, most CRC patients who choose XELOX-treated were more likely in an advanced cachexia state, which was similar to the reason for the T4 stage.

The findings outlined above suggest that high skeletal muscle mass may be associated with an increased risk of disease progression and mortality in patients with T4 nonmetastasis CRC patients. Moreover, the significance of these relationships is also significant in postoperation patients treated with XELOX. These results suggest that assessing skeletal muscle mass may be worthwhile when selecting treatments for CRC.

Nomogram is an alignment chart composed of lines of different proportions, which generates a total point to predict the likelihood of clinical events [26]. Quantify the relative contribution of each prognostic factor and convert complex regression models into visual graphics, which is more practical and convenient for evaluating the prognosis of CRC patients. According to the construction of the CT-based nomogram, some effective and targeted visceral fat and skeletal muscle mass interventions should be taken to reduce recurrence and prolong overall survival in CRC patients underwent surgery and regularly standard chemotherapy [27].

However, there are several limitations to our study. Firstly, CRC patients, without abdominal CT scans before curative surgery, were excluded. Besides, our study was comprised of a limited number of CRC patients; in particular, the diametrically inverse results in the T2-3 and T4 subgroups were found. Therefore, it will be more accurate and meaningful to establish quantified nomograms for OS and PFS if the number of samples is larger. In addition, these factors, such as correction of height, measurement of muscle density, and exclusion of fat infiltration in the muscle, had not been taken into consideration by us. Meanwhile, combined preoperative CT-quantified body component measures with muscular physical function measures will better predict the prognosis of CRC patients in the early stage. Another shortcoming of our research was the lack of regular follow-up CT scans during chemotherapy, and body composition measures were not available to verify the hypothesis.

## 5. Conclusions

CT-quantified body compositions have a significant influence on CRC patients successively underwent curative resection and regularly standard chemotherapy with the endpoints of 1-year, 3-year, and 5-year both OS and PFS. Patients with high SM showed a strikingly poor prognosis in OS and PFS in the T4 subgroup; however, the prognosis role of body composition was totally opposite in T2-3 patients.

## Figures and Tables

**Table 1:** Baseline patient characteristics.

	All	Xelox (*N* = 179)	Folfox/Folfiri (*N* = 42)	*P* value
Characteristics	Patient (%)			
Age (range)				0.297
Median (range)	60.98 ± 12.50	60.66 ± 12.45	61.17 ± 16.29	
<50	45 (20.3%)	34 (19.0%)	11 (26.2%)	
≥50	176 (79.7%)	145 (81.0%)	31 (73.8%)	
Gender				0.161
Male	129 (53.1%)	103 (57.5%)	26 (63.4%)	
Female	92 (46.9%)	66 (42.5%)	15 (36.6%)	
T stage				0.173
T2-3	116 (19.5%)	93 (52.0%)	23 (54.8%)	
T4	105 (80.5%)	76 (48.0%)	19 (45.2%)	
Lymph node metastasis				0.135
No	138 (56.3%)	116 (64.8%)	22 (52.4%)	
Yes	86 (43.7%)	63 (35.2%)	20 (47.6%)	
TNM stage				0.197
I-II	130 (54.5%)	109 (60.9)	21 (50.0)	
III	91 (45.5%)	70 (39.1)	21 (50.0)	
BMI				**<0.001**
<18.5	12 (5.4%)	8 (4.5%)	4 (9.5%)	
18.5-25	168 (76.0%)	135 (75.4%)	33 (78.6%)	
≥25	41 (18.6%)	36 (20.1%)	5 (11.9%)	
MetS				0.893
No	196 (88.7%)	159 (88.8%)	37 (88.1%)	
Yes	25 (11.3%)	20 (11.2%)	5 (11.9%)	
ECOG				**<0.001**
1	77 (34.8%)	71 (39.7%)	6 (14.3%)	
2	119 (53.8%)	99 (55.3%)	20 (47.6%)	
>3	25 (11.3%)	9 (5.0%)	16 (38.1%)	
Status				
Alive	156 (79.1%)	133 (74.3)	23 (54.8%)	
Death	65 (20.9%)	46 (25.7)	19 (45.2%)	

## Data Availability

All of the patient CT-quantified body composition data were uploaded in the supplemental file.

## Supplementary Materials

Supplementary Materials Supplementary 1 Figure S1: outcomes T2-T3 CRC patients based on CT body composition. Outcomes based on visceral fat in T2-T3 CRC patients from the time of diagnosis. (a) Overall survival. (b) PFS. Outcomes based on skeletal muscle in T2-T3 CRC patients from the time of diagnosis. (c) Overall survival (d) PFS. Supplementary 2 Figure S2: outcomes CRC patients treated with XELOX based on CT body composition. Outcomes based on visceral fat in CRC patients treated with XELOX. (a) Overall survival. (b) PFS. Outcomes based on skeletal muscle in CRC patients treated with XELOX. (c) Overall survival (d) PFS. Supplementary 3 Figure S3: outcomes CRC patients treated with FOLFOX/FOLFIRI based on CT body composition. Outcomes based on visceral fat in CRC patients treated with FOLFOX/FOLFIRI. (a) Overall survival. (b) PFS. Outcomes based on skeletal muscle in FOLFOX/FOLFIRI CRC patients. (c) Overall survival (d) PFS. Supplementary 4 Table S1: correlation of VAT and body mass index. Supplementary 5 Table S2: correlation of SM and body mass index. Supplementary 6 The data registered the CRC patient CT-quantified body composition.
