# Supplementary material for: Association between CT-Quantified Body Composition and Recurrence, Survival in Nonmetastasis Colorectal Cancer Patients Underwent Regular Chemotherapy after Surgery
Source: Biomed Res Int. 2021 Mar 25;2021:6657566. doi: 10.1155/2021/6657566 (PMC8016588; doi:10.1155/2021/6657566)
Supplement: Supplementary Materials — Supplementary 1 Figure S1: outcomes T2-T3 CRC patients based on CT body composition. Outcomes based on visceral fat in T2-T3 CRC patients from the time of diagnosis. (a) Overall survival. (b) PFS. Outcomes based on skeletal muscle in T2-T3 CRC patients from the time of diagnosis. (c) Overall survival (d) PFS. Supplementary 2 Figure S2: outcomes CRC patients treated with XELOX based on CT body composition. Outcomes based on visceral fat in CRC patients treated with XELOX. (a) Overall survival. (b) PFS. Outcomes based on skeletal muscle in CRC patients treated with XELOX. (c) Overall survival (d) PFS. Supplementary 3 Figure S3: outcomes CRC patients treated with FOLFOX/FOLFIRI based on CT body composition. Outcomes based on visceral fat in CRC patients treated with FOLFOX/FOLFIRI. (a) Overall survival. (b) PFS. Outcomes based on skeletal muscle in FOLFOX/FOLFIRI CRC patients. (c) Overall survival (d) PFS. Supplementary 4 Table S1: correlation of VAT and body mass index. Supplementary 5 Table S2: correlation of SM and body mass index. Supplementary 6 The data registered the CRC patient CT-quantified body composition. [file 6657566.f1.zip › supplementary tables revision (1).pdf]

Table S1 Correlation of VAT and Body mass index

	ECOG-1	ECOG-2	ECOG-3	P-Value
T2-3	8.72±4.48	9.26±4.22	6.55±4.55	0.3544
T4	10.15±4.54	8.98±5.68	11.05±5.66	0.3644
	Mets 0	Mets 1-2	Mets 3-5	P-Value
T2-3	9.66±4.02	8.41±4.47	9.33±4.36	0.4554
T4	9.37±3.87	9.95±5.25	10.58±6.10	0.8534

Table S2 Correlation of SM and Body mass index

	ECOG-1	ECOG-2	ECOG-3	P-Value
T2-3	7.46±2.65	6.33±2.46	5.56±2.25	0.04125
T4	6.35±2.41	6.47±2.45	6.32±3.41	0.9739
	Mets 0	Mets 1-2	Mets 3-5	P-Value
T2-3	6.72±2.90	6.57±2.24	3.63±1.84	0.022
T4	6.23±2.65	6.26±2.55	6.81±2.68	0.2061
